# Prospective Distractor Information Reduces Reward-Related Attentional Capture

**DOI:** 10.5334/joc.375

**Published:** 2024-06-17

**Authors:** Justin Mahlberg, Daniel Pearson, Mike E. Le Pelley, Poppy Watson

**Affiliations:** 1 Monash University, Melbourne, Australia; 2 University of Sydney, Australia; 3 UNSW Sydney, Australia; 4 University of Technology, Sydney, Australia

**Keywords:** attention, reward, expectations, information seeking, incentive salience

## Abstract

Motivationally salient stimuli, such as those associated with reward, can automatically gain attentional prioritisation – even when individuals are motivated to ignore such stimuli. This ‘attentional bias for reward’ has often been interpreted as evidence for involuntary Pavlovian ‘sign tracking’ behaviour. The prioritisation of reward-signalling distractors may additionally reflect a drive to gain information about the state of the world, irrespective of the particular reward that is being signalled. In the current study we assessed whether forewarning participants on each trial as to the upcoming features of a distractor would reduce reward-related attentional capture. This manipulation reduces the information provided by the distractor, without affecting the magnitude of the signalled reward. Using eye tracking in Experiment 1, we found that reward-related attentional capture was virtually eliminated when participants were informed of the upcoming distractor colour (relative to the baseline condition when no information was provided). In Experiment 2, using a response-time version of the task, we again found a significant reduction in reward-related attentional capture when participants received information about the colour of an upcoming distractor, or information about the value of the upcoming reward. Finally, in Experiment 3 we assessed whether participants were using the pre-trial information to strategically inhibit attention to the upcoming distractor colour. The results of these experiments are discussed within the context of information-seeking accounts of reward-related attentional capture effects.

## Introduction

A wealth of evidence has demonstrated that individuals often find it challenging to ignore task-irrelevant yet salient stimuli. Such salient stimuli are distractors that stand out either by virtue of physical characteristics (such as colour or luminance; Theeuwes, 1992) or when they have been previously associated with motivationally salient outcomes (such as rewards or punishments; Anderson et al., 2011; Le Pelley et al., 2015; Mikhael et al., 2021; Nissens et al., 2017; Pearson et al., 2015).

Reward-related attentional biases have been widely reported using a variety of different procedures (Failing et al., 2015; Le Pelley et al., 2015; Anderson et al., 2011; Hickey et al., 2010; Mine & Saiki, 2015; see for reviews: Anderson, 2021; Watson, Pearson, Wiers, et al., 2019; Le Pelley et al., 2016). In the visual search task used by Pearson et al. (2015), a variant of which was used in the current study, participants earned reward by making an eye movement (saccade) directly to a shape singleton (a diamond target amongst five circles). All shapes were grey, other than one of the circles which was rendered in either blue or orange. The colour of this salient *distractor* circle signalled whether a high or low monetary reward could be earned for a rapid saccade to the target on that trial (with colour-reward contingencies counterbalanced across participants); however, participants were informed that if they ever looked at the coloured circle, the reward would be cancelled on that trial. The critical finding was that participants were counterproductively more likely to look at the distractor signalling availability of high reward than the distractor signalling low reward, even though this meant that they missed out on more of the high rewards (Pearson et al., 2015).

Reward-related attentional capture effects can be interpreted within an incentive salience framework whereby stimuli that are predictive of reward become themselves imbued with motivational salience via a dopamine-dependent process (Anderson et al., 2016; Berridge, 2007; Colaizzi et al., 2020; Flagel et al., 2007). In the specific value-modulated attentional capture (VMAC) protocol described above, where participants are never required to attend to or respond to the colours that signal available reward (cf. Anderson et al., 2011), the distractor colours are Pavlovian signals of reward. The involuntary orienting to these stimuli has thus been frequently likened to ‘sign-tracking’ behaviour observed in non-human animals (Colaizzi et al., 2020). Sign tracking refers to the tendency of some animals to approach and engage with a Pavlovian signal of reward (e.g., a light that signals food delivery) rather than increasing approach towards the food magazine where the food reward is actually delivered (Flagel et al., 2007; Hearst & Jenkins, 1974). As in the VMAC task described above, animal studies have demonstrated that sign-tracking behaviour can be maladaptive, persisting even if it results in the loss of the signalled reward (Hearst & Jenkins, 1974).

The coloured distractors in the VMAC task are not just predictive of reward, they also have informational value. That is, the presence of a particular colour is perfectly diagnostic as to whether the current trial is a high-reward or low-reward trial, and thus has the potential to reduce participants’ degree of uncertainty about the current state of the world. Based on observations that information is often sought out by humans and animals even when it is of no instrumental value, prominent theories of attention and decision making argue that uncertainty-reduction acts as a primary driver of attention, favouring diagnostic cues (Gottlieb, 2012, 2018; Gottlieb et al., 2020; Gottlieb & Oudeyer, 2018). Such an ‘information gain’ mechanism is argued to be distinct from effects of reward prediction (Bromberg-Martin & Monosov, 2020; van Lieshout et al., 2020), with effects of both on attentional prioritisation (Cogliati Dezza et al., 2023; FitzGibbon et al., 2020; Gottlieb, 2018; Gottlieb & Oudeyer, 2018). Although these effects are difficult to disentangle, recent neuroimaging studies have reported distinct patterns of neurons that encode either expected reward or expected information gain (Cogliati Dezza et al., 2022; Filimon et al., 2020; Horan et al., 2019) suggesting that both are important in guiding attention for action.

Reward-related attentional bias (as measured with the VMAC task) is clearly influenced by reward prediction, because distractors signalling high reward capture attention more than distractors signalling low reward, even though the information gain signalled by the two distractors is equivalent (Gottlieb & Oudeyer, 2018). The question remains however as to whether information seeking also contributes to the VMAC effect, or whether an incentive-salience account can fully account for attentional prioritisation of distractors.

In the current study, we assessed evidence for information seeking in the VMAC task by providing participants with advance information on the type of distractor (and hence reward magnitude) that would be available on the upcoming trial. Since participants already knew the reward that would be available, this manipulation eliminated the information gain that would otherwise be provided by the subsequent presentation of the distractor. If the VMAC effect in this task is driven by information gain, then provision of instructions should reduce or eliminate this effect, relative to the standard (non-instructed) condition. By contrast, if the VMAC effect reflects sign-tracking based on the magnitude of the signalled reward, then it should be unaffected by the instruction manipulation.

Only one previous study has attempted to examine whether attentional capture by reward-associated stimuli would be impacted by providing participants with pre-trial information as to the magnitude of reward that was available (Failing & Theeuwes, 2017). In Experiments 1 and 3 of Failing and Theeuwes (2017), participants heard a tone during the pre-trial fixation period, and the frequency of the tone (high or low) indicated that high or low reward could be earned by a quick and correct response in the upcoming trial. The trials contained a search array with six outline circles with unique colours, with one coloured circle (e.g., green) containing the target. The colour of one of the non-target (distractor) circles signalled the reward available on the current trial, e.g., a blue distractor might signal high reward and a red distractor might signal low reward. The authors reported a reversed-VMAC effect: participants responded faster on trials featuring a high-reward relative to a low-reward distractor. However, in their procedure: (1) participants were never explicitly informed of the relationship between distractor colours and reward; (2) up to the critical measurement phase of the experiments, the colour had *always* been a redundant predictor of reward, since pre-trial instruction was always provided; and (3) the reward-associated distractor stimuli were not physically salient – all items were rendered in unique colours, so none was particularly distinctive. Together these aspects raise the possibility that participants may never have learned the relationship between colours and reward: in effect, the pre-trial instruction may have blocked learning about the significance of the low-salience distractor colours (Kamin, 1968), so that they never became perceived as information-providing signals of reward (no measure of colour–reward contingency learning was taken, which would have allowed this possibility to be assessed).

It thus remains an open question as to whether attentional capture by reward-associated stimuli could be moderated by prior knowledge of the upcoming distractor. To investigate this, Experiment 1 used a modified VMAC task, similar to that used by Pearson et al. (2015). We included trials that provided information about the distractor type featured in the upcoming trial, as well as control trials that simply featured a generic warning that the trial was about to commence. These generic-warning trials allowed participants an opportunity to experience the colour-reward contingencies in the absence of expectancies as to which distractor was going to appear and provided a baseline measure against which we could investigate the effect of the pre-trial information on visual search performance.

## Experiment 1

In Experiment 1, we used eye tracking to investigate whether attentional capture by reward-related distractors would be reduced when participants knew which distractor to expect on each trial. We compared performance on trials where participants had knowledge of the upcoming distractor type (e.g., blue/high reward or orange/low reward; counterbalanced across participants) to trials where they received generic instructions that the trial was about to begin. We were primarily interested in whether pre-trial instructions would reduce the VMAC effect, so we compared attentional capture by the coloured distractor on high reward vs. low reward trials (as a function of instruction type).

### Method

#### Participants and Apparatus

This study was approved by the UNSW Sydney Human Research Ethics Advisory Panel (Psychology) with file number HREAP 3503. We recruited for as many days as were necessary to test at least 33 participants, since G*Power analysis indicated that this would give 80% power to detect a medium effect size (d_z_ = 0.5) for the critical comparison of whether the VMAC effect was reduced in the informed vs. non-informed condition (two-tailed t-test). Our final sample comprised 35 student participants (25 women; age *M* = 18.75 years, *SEM* = 0.35 years) who participated for course credit. Participants could also earn a performance-contingent bonus during the task (ranging from $5–$12). No participants were excluded from the analysis.

Participants were tested individually, with head position stabilised using a chin rest 60 cm from the screen. Gaze was recorded using a Tobii Pro Spectrum eye-tracker (sample rate 600 Hz) mounted on a 23-inch monitor (1920 × 1280 resolution, 120 Hz refresh rate). Where stimulus presentation was gaze-contingent, the gaze data was first down-sampled to 100 Hz for online calculations. The eye tracker was calibrated at the start of the search task.

#### Design

**Figure 1:**
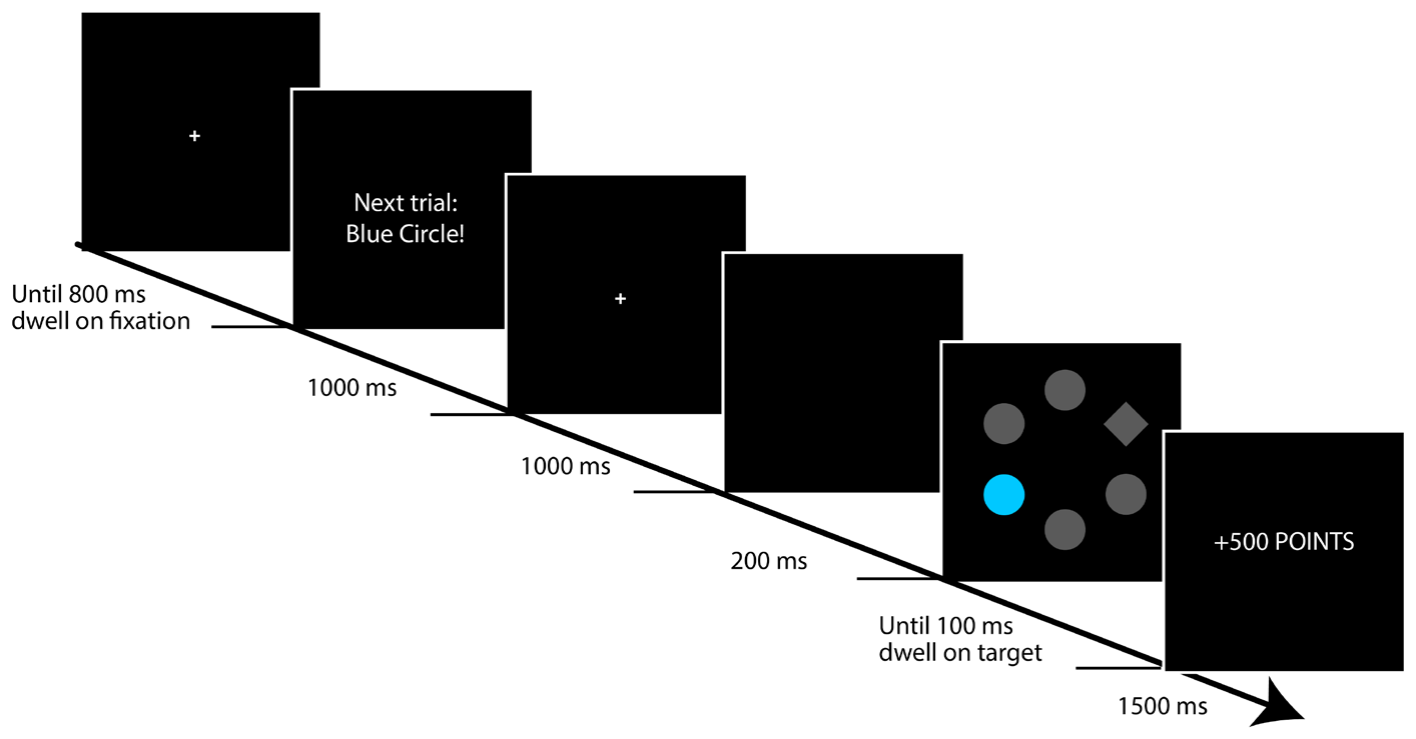
Trial structure of the instructed visual-search task used in Experiment 1. Participants’ task was to move their eyes to the diamond target as quickly and directly as possible to earn points (and money). The colour of the distractor (blue or orange; counterbalanced) signalled whether 500 or 10 points were available, but reward was omitted if participants looked at the distractor. The search display was preceded by an instruction screen either informing the participant of the upcoming distractor colour (‘informative trials’) or with a generic instruction that the trial was about to start (‘general trials’).

All stimuli were presented on a black background. Each trial of the search task began with a central, white fixation cross inside a white circle 2.6° visual angle: see Figure 1). After 800 ms of gaze had accumulated within the circle, or after 3000 ms, pre-trial instructions appeared for 1000 ms in white font, either providing a general warning about the upcoming trial (“Next trial: Coming up!”) or providing information on the distractor that would be presented on the upcoming trial (“Next trial: Blue circle!”, “Next trial: Orange circle!”, or “Next trial: No coloured circle!”). The fixation cross then reappeared; after 1000 ms the screen blanked for 200 ms before the search display appeared.

The search display consisted of six shapes – five circles and the diamond target (each 2.3 × 2.3° visual angle). The shapes were evenly distributed around the centre-point of the screen, with the centre of each shape at an eccentricity of 5.1°. All shapes were grey (RGB: 70, 70,70; luminance ~8.3 cd/m^2^) apart from one of the circles (the distractor) which was rendered on most trials in either orange (RGB: 193, 95, 30) or blue (RGB: 37, 141, 165) with similar luminance (~24.5 cd/m^2^). On infrequent ‘distractor-absent’ trials, all shapes were grey and one of the grey circles functioned as distractor. Target and distractor location were determined randomly on each trial with the constraint that the distractor was either next to, or two places away from the target in either direction (clockwise or anticlockwise), i.e., the distractor never appeared directly opposite the target. Trials terminated when a response was registered or after 2000 ms (timeout). For gaze-contingent calculations a small area of interest (AOI) with radius 1.75° visual angle was centred on the target, and a larger AOI (radius 2.55°) was centred on the coloured distractor. These sizes ensured that all AOIs were non-overlapping.

Participants’ task was to move their eyes to the diamond target ‘as quickly and directly as possible’; a response was registered when 100 ms of gaze had accumulated in the target AOI. The colour of the distractor circle indicated how many points were available on the current trial, for a rapid eye movement to the target (points were later converted to a monetary bonus). For half of the participants, a blue distractor signalled that 10 points could be earned while an orange circle indicated that 500 points were available. This colour-reward mapping was reversed for the other half of the participants. If any gaze was registered in the distractor AOI, the reward was omitted and the trial was recorded as an *omission trial*. On trials that did not feature a colour-singleton distractor (distractor-absent trials), participants could earn 10 points for a rapid eye movement to the target. Any gaze on the grey circle that was designated as distractor resulted in the trial being recorded as an omission trial. In distractor-absent trials, participants rarely looked at the grey circle that had been randomly designated as leading to reward omission: proportion of reward omissions for distractor-absent trials was *M* = 0.04, *SEM* = .008. We do not analyse data from distractor-absent trials further here: our focus in this study was on reward-related attentional capture (i.e., how attentional capture by distractors differed as a function of the size of the reward they signalled), so our analyses focused on data from trials containing a reward-signalling distractor in the search display. Nevertheless, we note that raw data for all trial types are available at https://osf.io/w65f2/.

Feedback was presented for 2500 ms during the first block of the experiment and for 1500 ms thereafter. If the trial was an omission trial then feedback showed “ +0 points”. If the trial was not an omission trial and a response was registered within 1000 ms then feedback stated how many points the participant had won on that trial: “+500 points” on trials with a high-reward distractor, and “+10 points” on trials with a low-reward distractor or distractor-absent trials. If response time was greater than 1000 ms, feedback read “+0 points, too slow”. If the trial timed out with no response, feedback read: “Too slow, please try to look at the diamond more quickly”. The ITI was 1200 ms.

Trials were arranged in blocks of 40, comprising 20 trials with pre-trial distractor information and 20 with general pre-trial information. Of the 20 trials of each information type, 8 featured a high-reward distractor, 8 featured a low-reward distractor and 4 had no colour-singleton distractor in the display (distractor-absent trials). Trial order was selected at random.

#### Procedure

Instructions at the start of the task informed participants that they could earn points on each trial by moving their eyes quickly and directly to the diamond target, and that they would receive a monetary bonus that would “typically be between $6 and $10” depending on how many points they earned (no information on the conversion rate between points and money was given). Participants were also told about the relationship between distractor colour and reward (e.g., that if the search array contained a blue distractor they could win 500 points, and if it contained an orange distractor they could win 10 points). Instructions emphasised that participants’ task was to look at the diamond, and if they looked at the coloured circle before looking at the diamond they would receive no reward, “so you should try to move your eyes straight to the diamond”. Check questions were used to verify that participants had understood these instructions.

To familiarise themselves with the task, participants first completed one block of pre-training (40 trials). Trials in the pre-training phase were as described above, but they did not contain the 1000-ms instruction screen (data from the pre-training phase were not analysed). Following the pre-training phase, participants were told that for the remainder of the task each trial would begin with an instruction screen that would sometimes tell them about the colour of the circle in the upcoming search array, and that they could use this information to prevent themselves from looking at the coloured circle and therefore earn more points. Participants then completed six blocks of forty trials with a self-paced break after each block (240 trials total). On completion of the task participants were informed of the amount of money they had earned and received their bonus.

#### Data Processing

For all experiments, the raw and processed means data for each participant are available at: https://osf.io/w65f2/.

Processing of data from the instructed phase of the task followed our standard procedures (e.g., Le Pelley et al., 2019; Pearson et al., 2016). We discarded the first two trials of each block, trials with less than 25% valid gaze location data (0.06% of all trials) and trials that timed out with no response registered (0.8% of all trials). In this task, our primary dependent variable was the proportion of omission trials, i.e., trials in which gaze was detected on the coloured distractor leading to cancellation of the signalled reward. The proportion of omission trials was calculated separately for trials that had the general “trial coming up” instruction (*general trials*), and for trials that were preceded by valid information about the upcoming distractor type (*informative trials*) as a function of distractor type: high-reward distractor vs. low-reward distractor. In addition, given that previous studies have reported reduced distraction across blocks of repeated distractor colours (e.g., Gaspelin et al., 2019; Vatterott & Vecera, 2012), we included block (1–6) in the analysis. We used ANOVA to examine the effect of instructions on the VMAC effect (high vs. low reward trials) and complemented critical t-test comparisons with the corresponding Bayesian t-tests, to examine the strength of evidence for the alternative vs. null hypotheses (using the criteria of Lee & Wagenmakers, 2013). All t-test comparisons are two-sided.

To investigate whether pre-trial information about the upcoming distractor type influenced how quickly participants began moving their eyes upon presentation of the search display, we also investigated the latency of first saccades on non-omission trials (i.e., trials on which participants did not look at the distractor). For this latency-based analysis, gaps in the raw gaze data of less than 75 ms were linearly interpolated and the data were smoothed with a five-point moving average filter. We then used a velocity-threshold identification algorithm (Salvucci & Goldberg, 2000) to identify the latency of the first saccade on each trial, defined as the duration from onset of the search display to the first time at which eye-movement velocity exceeded 40° visual angle per second for at least 10 ms. The saccade was recorded as going towards the target if the saccade vector had an angular deviation less than 30° to the left or right of the centre of the target (see Pearson et al., 2016). In addition to the trial-level exclusions noted in the previous paragraph, we excluded trials from the latency-based analysis if the saccade latency was below 80 ms (suggesting an anticipatory saccade), if there was insufficient gaze data to identify a saccade, or if the saccade starting point was more than 100 pixels from the centre of the screen. We excluded seven participants from the saccade analysis because they had more than 20% of total trials excluded on this basis (in line with our previous work e.g., Watson et al., 2022). For the remaining 28 participants, an additional 3.6% of total trials were discarded based on these criteria. For each participant and for each of the six trial types (2 instruction conditions × 3 distractor types) we then calculated the mean saccade latency for the subset of trials where this first saccade went towards the target.

## Results

### Omission Trials: Effect of Reward

**Figure 2:**
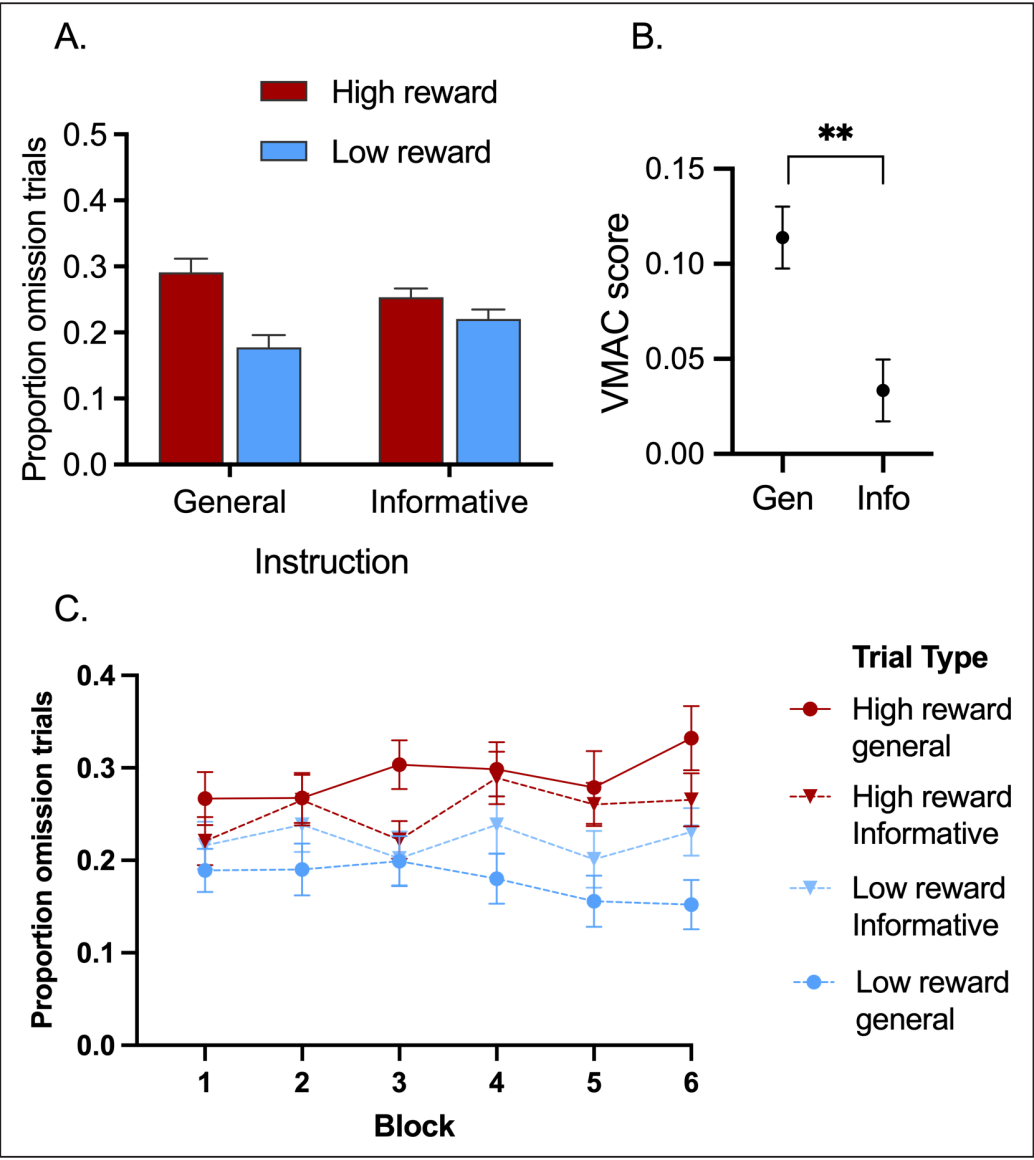
**Performance in Experiment 1 A.** Proportion of omission trials is shown as a function of instruction type (informative or general) and distractor type (high-reward or low-reward distractor). Omission trials are those where participants’ gaze was registered on the distractor leading to omission of reward. **B.** The value-modulated attentional capture effect (difference in proportion of omissions on high-reward relative to low-reward trials) is shown separately for trials with informative instructions as to the upcoming distractor colour (‘Info’ trials) and trials with a generic message that the trial was going to begin (‘Gen’ trials). **C.** Proportion of omission trials across blocks. Error bars represent within-subject SEM (Cousineau, 2005; Morey, 2008). ** = *p* < 0.01.

Figure 2a shows the mean proportion of trials where participants looked at the distractor, leading to omission of the signalled reward. To examine the effect of pre-trial instruction on distraction by reward-signalling stimuli (i.e., VMAC) we used repeated-measures ANOVA with factors of instruction type (general vs. informative), distractor type (high reward vs. low reward) and block (1–6). The main effect of instruction type was non-significant, *F* < 1, *p* = .786, *η_p_*^2^ = 0.002. More importantly, there was a significant main effect of distractor type, *F*(1,34) = 8.97, *p* = .005, *η_p_*^2^ = 0.21, with participants more likely to look at high-reward distractors than low-reward distractors. Critically this effect was moderated by an interaction with instruction type, *F*(1,34) = 12.27, *p* = .001, *η_p_*^2^ = 0.27, with the effect of distractor type being reduced by informative instructions relative to general instructions. Figure 2b highlights this significant interaction by showing VMAC scores (calculated as the difference in proportion of omissions on high-reward relative to low-reward trials) in each of the instruction conditions. A Bayesian *t*-test provided strong evidence for a difference in the VMAC effect between the informative and general trials, BF_10_ = 23.6. There was no significant main effect, nor interaction involving block, *F*s < 1.07, *p*s > .373, *η_p_*^2^s < 0.03 (see Figure 2c).

Returning to the data in Figure 2a, analysis of simple effects revealed that participants looked significantly less at the high-reward distractor on informed relative to general instruction trials *t*(34) = 2.26, *p* = .030. *d_z_* = 0.38, although the Bayes factor was anecdotal, BF_10_ = 1.34. Surprisingly, participants looked significantly *more* frequently at the low-reward distractor on informed relative to general instruction trials *t*(34) = 2.77, *p* = .009. *d_z_* = 0.82, with moderate evidence BF_10_ = 3.89.

#### First Saccade Latency to Target: Effect of Reward

**Figure 3:**
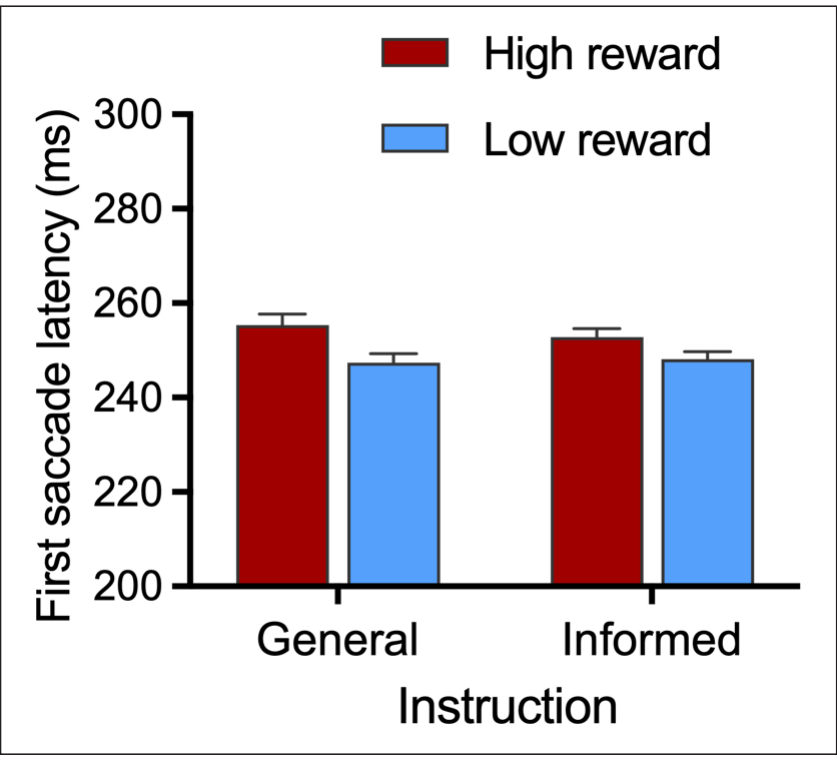
**First Saccade latencies to target in Experiment 1.** Trials where participants correctly identified the target without gaze being first registered on the distractor are shown as a function of instruction type (informative or general) and distractor type (high-reward or low-reward distractor). Error bars represent within-subject SEM (Cousineau, 2005) with Morey (2008) correction.

One potential explanation for reduced attentional capture by the high-reward distractor in the informative trials is that participants slowed down when they knew that a large reward was at stake (i.e. a speed-accuracy trade off). To examine whether pre-trial instructions influenced the speed at which participants began moving their eyes toward the target we examined first saccade latencies on trials where the eyes went directly to the target, using ANOVA with factors of instruction type (general vs. informative) and distractor type (high reward vs. low reward). As can be seen in Figure 3, participants were slower to move their eyes to the target on trials with a high-reward distractor versus a low-reward distractor, which was confirmed by a significant main effect of distractor type, *F*(1,27) = 7.41, *p* = .011, *η_p_*^2^ = 0.215. Moreover, the distractor effect was not impacted by whether a participant had information about the upcoming distractor type (main effect of instruction: *F* < 1, *p* = .503, *η_p_*^2^ = 0.02; interaction between distractor and instruction type: *F* < 1, *p* = .411, *η_p_*^2^ = 0.03).

Latencies of first saccades to target did not differ significantly between general relative to informed trials featuring a high-reward distractor, *t*(27) = .96, *p* = .348, *d_z_* = .18, with moderate evidence for the null hypothesis BF_01_ = 4.43, nor between general relative to informed trials featuring a low-reward distractor, *t*(27) = .38, *p* = .705, *d_z_* = .07, with moderate evidence for the null hypothesis BF_01_ = 6.39.

### Discussion

In Experiment 1 we used eye tracking to examine whether pre-trial information about the upcoming distractor colour would reduce attentional orienting to the distractor. On general baseline trials, in the absence of pre-trial information, we found a standard VMAC effect – increased attentional orienting to the distractor signalling high relative to low reward. However, when participants had knowledge of the upcoming distractor type prior to appearance of the search array, the VMAC effect was reduced to the point where there was no longer a significant difference in capture by high- versus low-reward distractors. Follow-up analyses suggested that the reduction in the VMAC effect was driven by a reduced tendency to look at the high-reward distractor on informative instruction trials, coupled with an increased tendency to look at low-reward distractors on informative instruction trials (the latter unexpected under an information-seeking account of VMAC; we return to this finding in the General Discussion). Importantly, the reduction in capture by high-reward distractors under informed conditions did not seem to reflect a strategic slowing of participants’ responses when they knew that high reward was available, in that latency to the first saccade was not greater when distractor information was provided. Instead, the implication is that pre-trial information rendered the high-reward distractor less likely to capture participants’ attention, without also impairing responses to the target.

Notably, the ability of the high reward distractors to capture attention on general information trials did not diminish across blocks, suggesting that the distractor retained its status as a predictor of reward throughout the task. That is, the introduction of informative instruction trials—rendering the information provided by the distractor redundant—did not impair the ability of the distractor to elicit conditioned behaviour when it was presented in the absence of pre-trial information (cf. the temporal primacy effect: Cole & McNally, 2007; Kehoe et al., 1987; Sutton & Barto, 1981). Thus, high-reward distractors retained the *potential* to elicit reward-related capture throughout the task, but instruction reduced the likelihood that they would do so.

## Experiment 2

**Figure 4:**
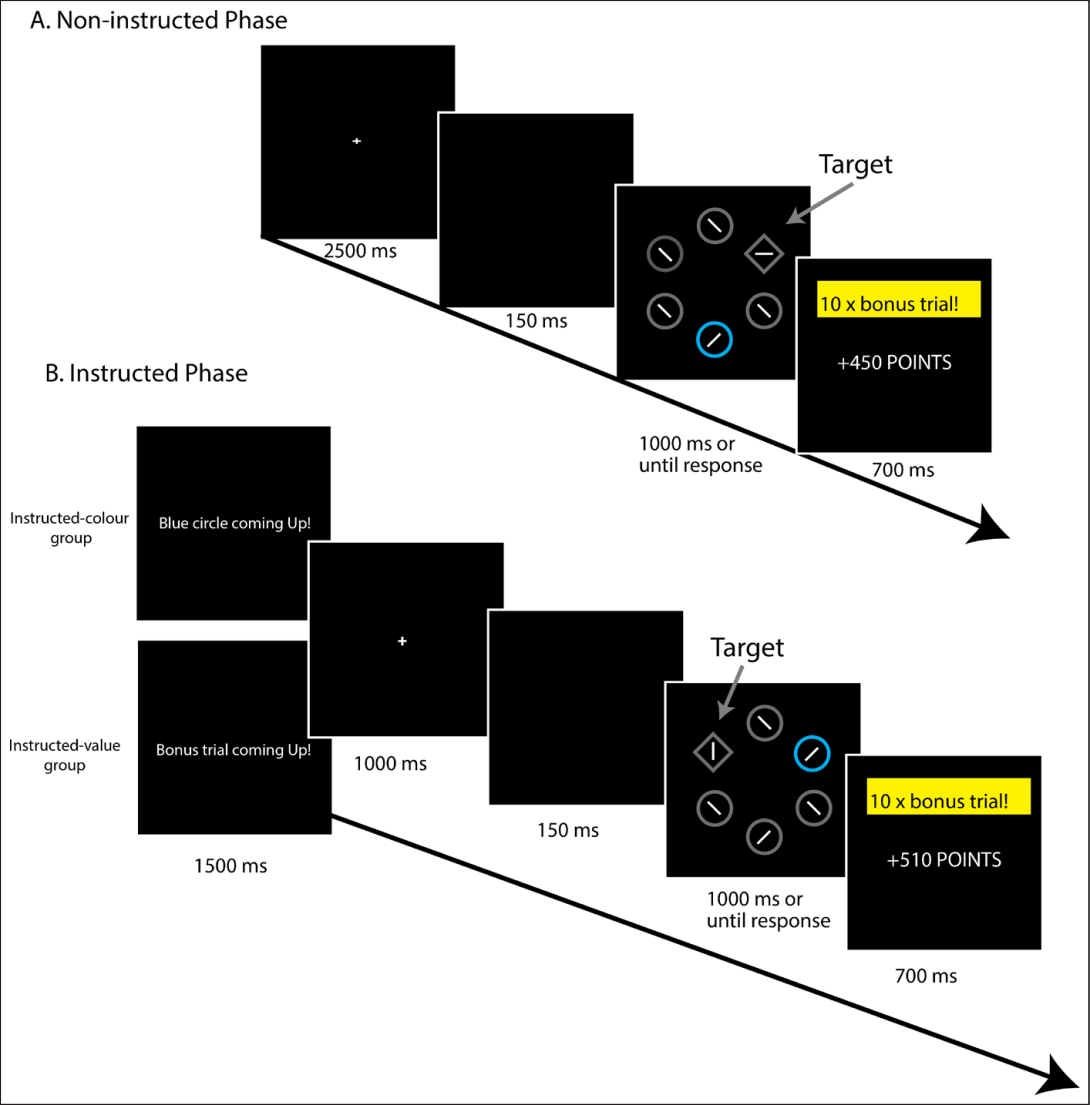
Trial structure during the RT version of the VMAC task used in Experiment 2. Participants’ task was to respond to the orientation of the line within the diamond. Faster responses earned more points with the colour of the distractor (blue or orange, counterbalanced) signalling whether points would be multiplied by 10 (bonus trials) or not (no-bonus trials). Participants first completed eight blocks of non-instructed trials **(A)** before completing five blocks of instructed trials **(B)** On instructed trials, the trial began with an instruction screen with a generic instruction that the trial was about to start ‘general trials’ or with an instruction informing the participant about the upcoming distractor ‘informative trials’. Participants in the instructed-colour group were informed about the upcoming distractor on informative trials whereas those in the instructed-value group were informed about whether the upcoming trial was a high-reward (bonus) trial or not.

The information-seeking account of the VMAC effect proposes that when participants were informed about the upcoming distractor type, the distractors had reduced informational value and were simply less distracting. Experiment 2 was designed to look at this information-seeking account in more detail. Due to the COVID pandemic we were unable to test participants in person, and hence could not use eye-tracking in Experiment 2. Consequently we moved to a response-time (RT) version of the task, which can be administered online (Albertella et al., 2019; Le Pelley et al., 2015, 2022; Watson, Pearson, Most, et al., 2019). Rather than the response being a saccade to the target, in the RT version of the task participants must make a button-press response as quickly as possible to the orientation of a line within the diamond target shape, with faster (correct) responses earning more points (see Fig 4). The colour of a distractor in the search display signals a reward multiplier: if the distractor appears in a high-reward colour, the trial is a bonus trial on which reward would be multiplied by a factor of 10; if the distractor appears in the low-reward colour, the trial was a standard (non-bonus) trial in which reward was delivered at the base rate. In the absence of eye-tracking we cannot directly assess whether participants have attended to the reward-signalling distractor on any given trial; instead in this version of the task, attention to the distractor is indirectly indexed by the degree to which presence of the distractor interferes with participants’ response to the target, on the assumption that if attention is captured by the distractor, this will slow responding to the target (Theeuwes, 1992, 1994). The typical finding is that responses to the target are slower (but not more accurate) when the display contains a high-reward distractor versus a low-reward distractor (Le Pelley et al., 2015, 2022; Watson, Pearson, Most, et al., 2019), suggesting that the high-reward distractor is more likely to capture attention and hence demonstrating a VMAC effect. As for the eye-tracking version of the task, this pattern of behaviour is counterproductive as it means that participants earn less on high-reward trials (when the largest rewards are potentially available) than would otherwise have been the case. This RT-based approach has been validated in meta-analysis as providing a reliable measure of the VMAC effect (Rusz et al., 2020).

In Experiment 2, we combined this RT-based version of the VMAC task with a manipulation of pre-trial information, as in Experiment 1. Specifically we adapted this task by implementing a between-subjects manipulation of the type of pre-trial information that was provided on informative trials. Participants in the instructed-colour group received information about the colour of the upcoming distractor. This group therefore provided a conceptual replication of Experiment 1. As noted above, the RT-based version of the task has previously been validated as a measure of VMAC, but including this replication of Experiment 1 within the design of Experiment 2 allowed us to verify that the critical finding—a reduction in attentional prioritisation by the high-reward distractor on colour-informed trials relative to uninformed trials—would also be obtained in this RT version of the search task (run online).

Going beyond the design of Experiment 1, participants in the instructed-value group of Experiment 2 instead received information about the available *reward* on the upcoming trial. If the reduction in VMAC under instructed-colour conditions was a consequence of pre-trial information rendering redundant the reward information provided by the distractor, then we should expect to see a similar reduction under instructed-reward conditions. While these instructions make different aspects of the upcoming trial (distractor colour or reward value) more readily available to participants, they are essentially equivalent because the colour of the upcoming distractor can be inferred from the reward and vice versa.

### Methods

#### Participants & Apparatus

To ensure enough power to be able to capture potential interactions between instructed group (instructed-colour vs. instructed-value groups) and VMAC scores in the two different conditions (general warning cues vs informative cues), we aimed to recruit at least 186 participants. A G*Power analysis indicated that this would give 90% power to detect a small effect size (f = 0.12) for the between/within F-test interaction (assuming correlation between repeated measures of 0.5 and sphericity correction of 1). We aimed therefore to test approximately 240 participants, assuming that data from 25–30% of the online sample would be excluded.

The task was programmed in jsPsych (de Leeuw, 2015) and 235 participants completed the study online in their own time. Participants were recruited from the online platform Prolific and earned £3, with the 20% of participants who earned the most points earning a bonus of £3. At the beginning of the experiment participants were randomly assigned to either the instructed-value group or the instructed-colour group.

#### Materials

Participants completed the RT version of the VMAC task online (see Figure 4). The task consisted of a non-instructed and an instructed phase. During the non-instructed phase (Figure 4A), the fixation display appeared for 2500 ms followed by a 150-ms blank screen. The search display was then presented for 1000 ms. This display consisted of six shapes (size: 100 px × 100 px) spaced evenly around the centre of the screen, with the centre of each shape at an eccentricity of 200 px. As in Experiment 1, the search display comprised five circles and one diamond (the target). Each circle contained a white line tilted 45° randomly to the left or right. The diamond target contained a line oriented either horizontally or vertically (randomly). On each trial, one of the circles (the distractor) was rendered in either blue or orange and all other shapes were grey. The colour of the distractor indicated whether it was a ’10 x bonus’ (high reward) or ‘standard’ (low reward) trial with the assignment of colour (blue/orange) to reward (high/low) counterbalanced across participants. Target and distractor location were randomly determined on each trial, with no constraints on distractor location. Participants responded as fast as possible to the orientation of the line within the diamond, pressing ‘C’ if it was horizontal and ‘M’ if it was vertical. Following the response (or after 1000-ms timeout) participants saw the feedback screen for 700 ms. Following an error the feedback screen read: “Error +0 Points”. If no response was recorded (time out), the screen read: “Too Slow. Please try to respond faster”. The feedback screen after correct responses displayed the number of points that had been won e.g., “+40 points”. For correct responses on low-reward trials participants earned 0.1 points for every ms that their response time was below 1000 ms (e.g., RT of 400 ms would earn 60 points). On high-reward trials the points were multiplied by 10 (e.g., RT of 400 ms would earn 600 points). Following (correct) high-reward trials, the feedback screen also displayed the text “10 x bonus trial!” in yellow. The ITI was 400 ms, 500 ms or 600 ms (selected at random).

Trials during the instructed phase of the task were the same as the non-instructed phase except that the trial began with a 1500 ms instruction screen followed by a 1000 ms fixation cross and a 150 ms blank screen (Figure 4B). The instruction screen either provided a general warning about the upcoming trial (“Next trial coming up!”) or it provided valid information as to the upcoming trial type. Participants in the instructed-colour group saw information as to the upcoming distractor colour (“Blue circle coming up!/Orange circle coming up!”). Participants in the instructed-value group saw information as to the upcoming distractor type (“Bonus trial coming up!/Standard trial coming up!”).

#### Procedure

At the beginning of the experiment all participants received general task instructions (to respond to the orientation of the line inside the diamond as quickly and accurately as possible) and were told the relationship between the coloured circles and the different trial types (e.g., orange signalled that any points earned would be multiplied by 10). They were also told that the 20% of participants with the most points would earn a £3 bonus, on top of the £3 base payment. Check questions had to be answered correctly before they could proceed. To ensure that participants had sufficiently learned the colour-reward contingencies before the critical instruction phase, they first completed eight blocks of non-instructed trials with a self-paced break every two blocks. Each block consisted of 18 trials (144 trials total in this phase). Half of the trials in each block featured the low-reward distractor, and the other half featured the high-reward distractor (in random order).

After completing the non-instructed phase, the initial instructions (to respond to the orientation of the line inside the diamond, the colour-reward contingencies and reminder that the 20% top-performing participants would receive a bonus) were repeated. In addition, participants read that “trials will now begin with instructions that will sometimes tell you what type of trial is coming up”. Check questions had to be answered correctly before they could proceed. Participants then completed five blocks of instructed trials, with each block consisting of 32 trials (160 trials total). Within each block, eight trials were generic instruction trials that were not informative as to the upcoming distractor type (four of the generic instruction trials featured a high-reward distractor and four featured a low-reward distractor). The other 24 trials in each block were informative trials (half high-reward distractor trials and half low-reward distractor trials) where participants received information about the upcoming trial type – either colour or reward, depending on group. At the end of the experiment participants were asked to report the associations between the distractor colours (orange and blue) and the trial types (standard trial and 10 x bonus trial).

#### Data Processing

We used separate ANOVAs to analyse data from the non-instructed phase of the task (when both groups received the same treatment) and the instructed phase of the task which had the additional factor of instruction type (general vs. informative). Processing of behavioural data followed our standard procedures for the RT version of the VMAC task (Le Pelley et al., 2022; Watson, Pearson, Most, et al., 2019). We discarded trials with responses that were either too slow (RT > 1000 ms) or anticipatory responses (RT < 150 ms). Analysis of RTs used correct responses only.

### Results

#### Participants

Forty-nine participants were excluded for having more than 20% invalid (anticipatory or timeout) trial exclusions or for having an error rate of more than 40% overall. One participant was excluded for answering both colour-reward contingency checks incorrectly at the end of the experiment. The remaining 185 participants had mean trial exclusions of 4.2% (SEM: 0.2%) and mean error rate of 15% (SEM: 0.5%). This final sample consisted of 93 participants in the instructed-colour group and 92 participants in the instructed-value group. Demographic data from one participant in the instructed-colour group was missing, but otherwise consisted of 47 females, 43 males, and two who identified as other (mean age: 26.48 years, SEM = 0.79). The instructed-value group consisted of 41 females, 50 males and one who identified as other (mean age: 27.59 years, SEM = 0.88). The instruction groups did not differ significantly in age, *t*(182) = 0.94, *p* = .349, *d* = .14 nor in distribution of males/females, χ^2^(1) = 0.79, *p* = .373.

#### Visual Search Performance: Non-instructed Phase

Mean error rates and RT in the non-instructed phase were examined via mixed ANOVA with factors of distractor type (high vs. low reward) and instruction group (instructed-colour vs. instructed-value group).

**Figure 5:**
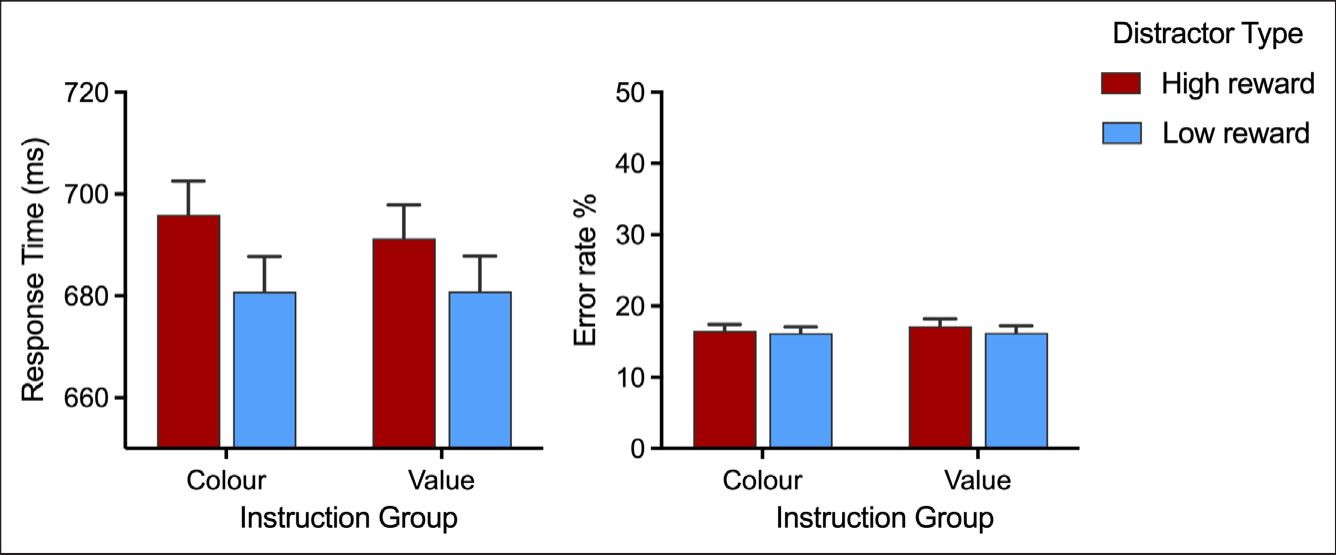
**Performance during the non-instructed phase of Experiment 2** as a function of distractor type (high reward vs low reward). Data shown separately for the instructed-colour and instructed-value groups. **Left Panel.** RT on correct trials. **Right Panel.** Error rates. Error bars represent SEM.

**RT.** The analysis of RTs revealed a main effect of distractor type, *F*(1, 183) = 56.80, *p* < .001, *η_p_*^2^ = 0.237, with slower responding on high-reward trials than low-reward trials (see left panel of Figure 5). This pattern demonstrates a VMAC effect during the non-instructed phase. Unsurprisingly given that the two instruction groups had so far had identical experiences, there was no main effect of instruction group, *F* < 1, *p* = .808, *η_p_*^2^ = 0.01, nor interaction between instruction group and distractor type, *F* (1,183) = 1.96, *p* = .164, *η_p_*^2^ = 0.01.

**Error Rates.** Analysis of error rates found no significant effects of distractor type, *F*(1,183) = 1.97, *p* = .162, *η_p_*^2^ = 0.01, instruction group, *F* < 1, *p* = .776, *η_p_*^2^ < 0.001, nor interaction between these two variables, *F* < 1, *p* = .486, *η_p_*^2^ = 0.003 (see right panel of Figure 5). Overall, then, participants were significantly slower but not more accurate on high-reward versus low-reward trials during the non-instructed phase, suggesting that the RT difference did not reflect a speed–accuracy trade off.

#### Visual Search Performance: Instructed Phase

Data from the instructed phase were analysed using mixed ANOVA with factors of distractor type (high vs. low reward), instruction type (informative vs. general), and instruction group (instructed-colour vs. instructed-value group).

**Figure 6:**
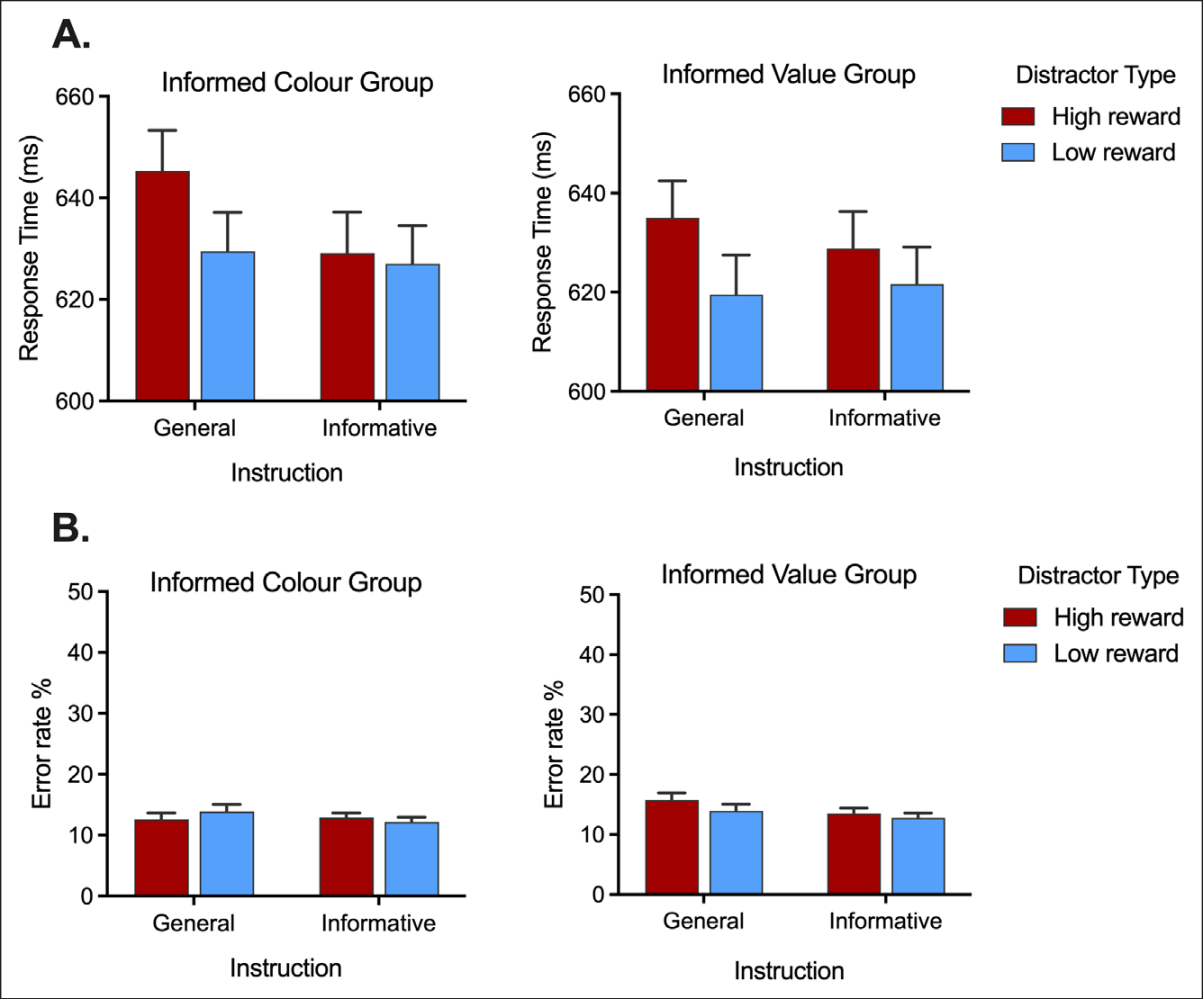
**Performance during the instructed phase of Experiment 2** as a function of instruction type (informative or generic) and distractor type (high-reward distractor and low-reward distractor), shown separately for participants in the informed-colour group (left panel) and the informed-value group (right panel). **A.** RT for correct responses. **B.** Error rates. Error bars represent *SEM*.

**RT.** Analysis of RTs (see Figure 6) revealed main effects of distractor type, *F*(1, 183) = 35.95, *p* < .001, *η_p_*^2^ = 0.16, and instruction type, *F*(1, 183) = 10.68, *p* = .001, *η_p_*^2^ = 0.06, that were superseded by a significant interaction between these two variables, *F*(1, 183) = 11.75, *p* = .001, *η_p_*^2^ = 0.06. Similar to Experiment 1, this interaction arose because the VMAC effect (i.e., the pattern of slower responding on high-reward than low-reward trials) was significantly reduced following informative instructions relative to general instructions. There was a significant interaction between instruction type and instruction group, *F*(1, 183) = 4.40, *p* = .037, *η_p_*^2^ = 0.02, because relative to the instructed-value group, the instructed-colour group responded slower overall in the general instruction condition and thus benefitted more from the informative instructions. There was no significant main effect of instruction group, *F* < 1, *p* = .538, *η_p_*^2^ = 0.002, nor was the three-way interaction significant, *F* < 1, *p* = .401, *η_p_*^2^ = 0.004.

Given the non-significant three-way interaction, we collapsed across the instructed-colour and instructed-value groups to investigate further the observed interaction between distractor type and instruction type. Overall, participants were significantly faster when high-reward trials were preceded by informative relative to general (non-informative) instructions, *t*(184) = 4.44, *p* < .001, *d_z_* = 0.32, BF_10_ = 500. There was no significant difference in RT on low-reward trials as a function of instruction type, *t*(184) = .08, *p* = .936, *d_z_* = 0.01; the Bayes factor indicated anecdotal evidence for the null BF_01_ = 1.46.

**Errors.** The mixed three-way ANOVA was repeated on the error rate data. Only the main effect of instruction type was statistically significant, *F*(1, 183) = 5.91, *p* = .016, *η_p_*^2^ = 0.03. Participants made fewer errors overall on the valid-instruction trials (mean: 12.8%, SEM: 5.4%) relative to the general-instruction trials (mean: 14.0%, SEM: 6.8%).

**Reporting of Colour-Reward Contingencies.** Overall accuracy was extremely high. All participants correctly identified that the high-reward colour signalled a 10 x Bonus trial. Nine participants (four from the instructed-colour group) incorrectly reported the association between the low-reward colour and the standard trials.

### Discussion

In Experiment 2 we provided participants with information about either the upcoming distractor colour (instructed-colour group) or the reward value/bonus status of the upcoming trial (instructed-value group). In a replication of the pattern of results from Experiment 1, we found that when participants had advance information about the type of trial – whether that was information about the upcoming distractor *colour* or the *reward value* – the magnitude of the VMAC effect was reduced relative to when no specific information was provided. More specifically, advance information led to a reduction in the likelihood that the reward-signalling distractor would capture attention (indexed here by RT to the target), on high-reward trials only. Notably, Experiment 2 demonstrated this pattern in an RT-based version of the VMAC task run online (as opposed to Experiment 1, which used an eye-tracking version of the task run in-person in the lab), underlining the reliability and generalisability of this finding.

Overall, this pattern of results could be interpreted to suggest that reduced distraction by the cue signalling high reward on informative trials is because the information provided by the distractor is now redundant (Gottlieb, 2012; Gottlieb & Oudeyer, 2018), rendering it less motivationally salient (Cogliati Dezza et al., 2023; FitzGibbon et al., 2020). To account for the fact that the effects of reduced information seeking are most visible on trials featuring a high-reward distractor, it would need to be assumed that information about the current trial type is particularly valuable on high- relative to low-reward trials – possibly because participants wish to ‘savour’ knowledge about the upcoming high-reward (Bromberg-Martin & Monosov, 2020; Kobayashi et al., 2019).

There is however an alternative explanation for the pattern of results in Experiments 1 and 2. Improved performance on informative high-reward trials may not be due to reduced salience of the distractor, but may instead reflect participants actively using information about the expected distractor colour to improve their search performance. We did not find any evidence for strategic slowing on high-reward informed trials on the eye tracking version of the task in Experiment 1, but another possibility is that participants are proactively inhibiting attention towards the high-reward distractor features, when they know to expect them. For example, if participants know that a blue circle is coming up, then they may be able to suppress attention to that feature in the display, and thus locate the target faster (de Vries et al., 2019; van Zoest et al., 2021). This would not be observed on low-reward trials, because reward salience is already minimal, and performance cannot be improved any further (Cunningham & Egeth, 2016; Mahlberg et al., 2023; Moher & Egeth, 2012; Wang & Theeuwes, 2018a).

## Experiment 3

Experiment 3 examined the hypothesis that reduced distraction on informative trials is due to participants using the pre-trial information to actively suppress attention to the high-reward distractor features in the display (de Vries et al., 2019; van Zoest et al., 2021). Such inhibition effects have been commonly observed in studies of spatial suppression, where across trials, the distractor repeatedly appears in one particular location (Le Pelley et al., 2022; Wang & Theeuwes, 2018b). Evidence for attentional suppression comes from occasional trials in which no distractor is presented, where it is found that responses to the target are significantly *slower* if it appears in this ‘frequent-distractor’ location versus any other location. Thus, to examine attentional suppression of colour in Experiment 3, we included rare trials where participants were expecting a coloured distractor (e.g., blue) but then unexpectedly we presented the target in that colour. Slowed target detection on trials where participants are expecting e.g., a blue distractor and the target appears in blue is a direct behavioural test of whether participants are using the knowledge about the upcoming distractor to proactively suppress stimuli rendered in that colour.

### Methods

#### Participants & Apparatus

G*Power analysis indicated that for the critical comparison of whether RT would be slower when the target appeared in the high-reward colour relative to when it was grey, 54 participants would give 95% power to detect a medium effect size (d_z_ = 0.5). In total, 61 participants completed the experiment via the online platform Prolific. The task was programmed in jsPsych (de Leeuw, 2015); participants completed the task in their own time, and earned £4, with the 20% of participants who earned the most points earning a bonus of £3.

#### Materials & Procedure

Participants completed the RT version of the VMAC task online (similar to the instructed-colour version of the task used in Experiment 2), with some minor changes. The non-instructed phase proceeded exactly as outlined in Experiment 2, but was shorter, with participants completing 4 blocks of 22 trials (88 trials total).

Trials during the instructed phase of the task were the same as the instructed-colour version of Experiment 2, except that all trials were informative (no general instruction trials were included) and on each trial participants were instructed about the colour of an upcoming *shape*, rather than a circle specifically (thus, “Blue shape coming up!/Orange shape coming up!”). To have a baseline against which to evaluate evidence for feature suppression effects, trials where all shapes were rendered in grey were also included (and always preceded by the instructions “No coloured shape coming up”). The first block consisted of 46 intermixed trials, 20 featuring a high-reward distractor, 20 with a low-reward distractor and 6 not featuring any coloured shapes. In addition to these 46 trials the subsequent four blocks contained an additional 10 trials (intermixed) where the target could appear in either the high- or low-reward colour (all other shapes were grey). Data processing followed procedures outlined in Experiment 2.

### Results

#### Participants

One participant was excluded for having more than 20% invalid (anticipatory or timeout) trial exclusions. No participants were excluded for having more than 40% errors. The remaining 60 participants had mean trial exclusions of 3.5% (SEM: 0.4%) and mean error rate of 13% (SEM: 1.0%). One person chose not to disclose demographic data, but the group otherwise consisted of 21 females, 37 males, and one who identified as non-binary (mean age: 32.8 years, SEM = 1.2).

#### Visual Search Performance: Non-instructed Phase

**Figure 7:**
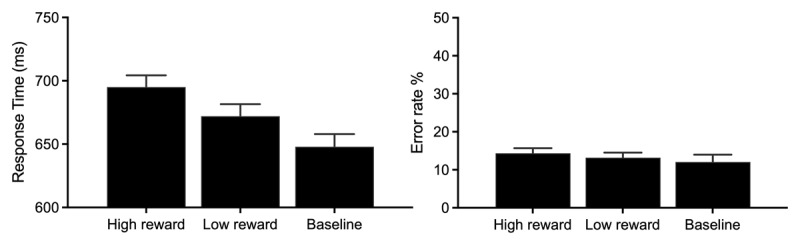
**Performance during the non-instructed phase of Experiment 3** on both high reward, low reward and baseline trials (all shapes rendered in grey). **Left Panel.** RT for correct responses. **Right Panel.** Error rates. Error bars represent within-subject SEM (Cousineau, 2005) with Morey (2008) correction.

Mean RT and error rates in the non-instructed phase are shown in Figure 7, and were examined via repeated measures ANOVA with factor of trial type (high reward, low reward, baseline trials where all shapes are grey).

**RT.** The analysis of correct RTs revealed a main effect of distractor type, *F*(2,118) = 58.34, *p* < .001, *η_p_*^2^ = 0.493, with slower responding on high-reward trials relative to low-reward trials indicative of a VMAC effect, *t*(59) = 5.56, *p* < .001, *d_z_* = .72, BF_10_ = 21,276. Responses on trials with a low-reward distractor were in turn slower than baseline trials where all shapes were rendered in grey, *t*(59) = 5.56, *p* < .001, *d_z_* = .72, BF_10_ = 22,727.

**Error Rates.** Analysis of error rates found no significant effect of trial type, *F*(2,118) = .93, *p* = .396, *η_p_*^2^ = 0.02.

#### Visual Search Performance: Instructed Phase

**Figure 8:**
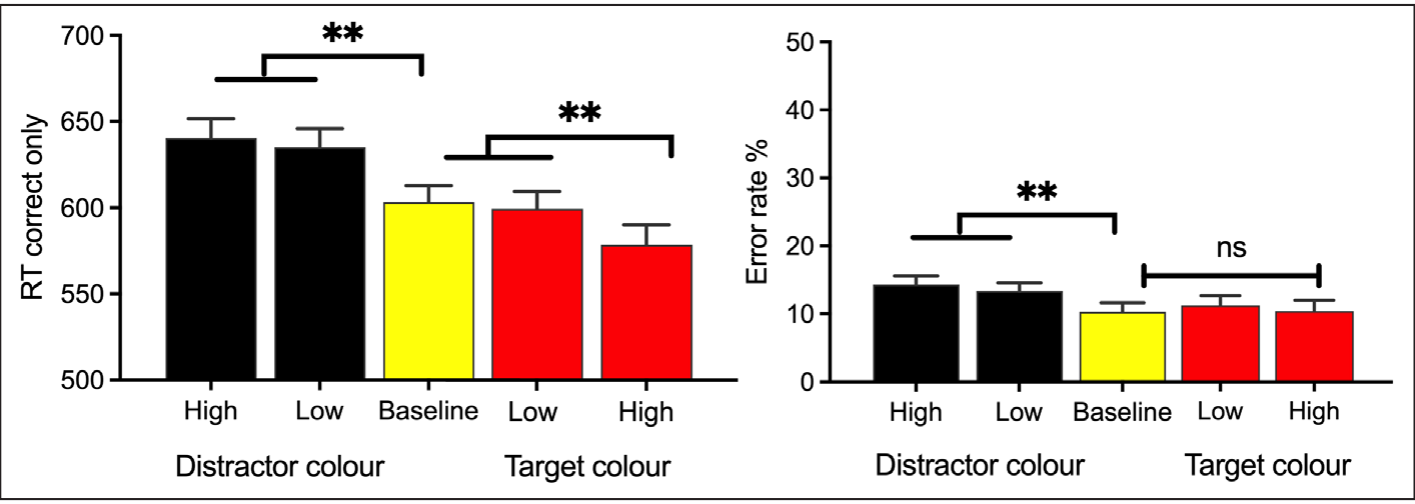
**Performance during the instructed phase of Experiment 3.** Trials where the distractor appeared in the cued colour (high reward or low reward) are depicted in black. Trials where the target unexpectedly appeared in the cued colour (High reward or Low reward) are depicted in red. Performance on baseline trials where all shapes were rendered in grey (yellow bar) was included in both analyses. **Left Panel.** RT for correct responses. **Right Panel.** Error rates. Error bars represent within-subject SEM (Cousineau, 2005) with Morey (2008) correction. ** p < .001.

Corresponding data from the instructed phase are shown in Figure 8. We first used repeated measures ANOVA to analyse performance on trials where the distractor appeared in the cued colour (distractor rendered in high reward colour, distractor rendered in low reward colour) relative to the baseline condition when all shapes were rendered in grey. We then repeated the analysis for trials where the *target* unexpectedly appeared in either the low-reward or high-reward colour and compared performance on these trials to the baseline condition.

**RT.** In the first repeated measures ANOVA we included three trial types: those where participants were forewarned of an upcoming shape in the high-reward or low-reward colour and the distractor appeared in that colour, and those where participants were forewarned that no coloured shape would appear (baseline). This analysis revealed a main effect of trial type, *F*(2,118) = 30.24, *p* < .001, *η_p_*^2^ = 0.34. As depicted in Figure 8 (left panel, black bars), there was no significant difference in RT between trials featuring a high-reward or low-reward distractor, *t*(59) = .87, *p* < .387, *d_z_* = .11, with moderate evidence for the null BF_01_ = 6.8 demonstrating that the pre-trial instructions virtually eliminated the VMAC effect that had previously been observed during the non-instructed phase. Significantly slower responses to the target were observed when the display contained a colour-singleton distractor than when it did not: low-reward-distractor vs baseline condition, *t*(59) = 9.26, *p* < .001, *d_z_* = .86, BF_10_ > 100,000.

In the second ANOVA analysis of RT we included three trial types: trials where participants were forewarned of an upcoming shape in the high-reward or low-reward colour and the *target* appeared in that colour, and trials where participants were forewarned that no coloured shape would appear (baseline). This analysis revealed a significant effect of trial type, *F*(2,118) = 11.12, *p* < .001, *η_p_*^2^ = 0.26. As depicted in Figure 8 (left panel, red bars), participants were significantly *faster* to respond to the target when it unexpectedly appeared in the high-reward relative to the low-reward colour, *t*(59) = 3.4, *p* < .001, *d_z_* = .44,with anecdotal evidence from the Bayes factor, BF_10_ = 1.85. There was no significant difference in RT when the target unexpectedly appeared in the low-reward colour, relative to the baseline condition where all shapes were grey, *t*(59) = 1.20, *p* = .214, *d_z_* = .16, with moderate evidence for the null hypothesis, BF_01_ = 4.6.

**Errors.** Analysis of error rates for trials where participants were forewarned of an upcoming shape in the high-reward or low-reward colour and the distractor appeared in that colour (or forewarned that no coloured shape would appear), revealed a main effect of trial type, *F*(2,118) = 13.07, *p* < .001, *η_p_*^2^ = 0.18. As can be seen in Figure 8 (right panel, black bars), there was no significant difference in error rates between trials featuring a high-reward or low-reward distractor, *t*(59) = 1.45, *p* < .153, *d_z_* = .19, but participants made significantly fewer errors on baseline trials (where all shapes were rendered in grey) relative to low-reward trials, *t*(59) = 3.67, *p* < .001, *d_z_* = .47.

Analysis of errors for trials where participants were forewarned of an upcoming shape in the high-reward or low-reward colour and the target appeared in that colour (or forewarned that no coloured shape would appear), revealed a non-significant effect of trial type, *F*(2,118) = .52, *p* = .597, *η_p_*^2^ = 0.01.

### Discussion

In line with the findings from the previous experiments the introduction of instructions in Experiment 3 eliminated the VMAC effect that had been observed during the non-instructed phase. Data from the occasional trials in which the target unexpectedly appeared in the high-reward or low-reward colour allowed us to test a distractor suppression account. If participants were using the pretrial information to strategically inhibit attention to the colour of the upcoming coloured shape, then RT should be slower on trials when the target was rendered in the high-reward colour (relative to all other conditions). However, we did not find any evidence for this and instead saw *faster* responses when the target appeared in the high-reward colour. These data do not accord with a proactive distractor suppression account (nor particularly well with an information-seeking account), the implications of which will be considered in the General Discussion.

## General Discussion

Using eye-tracking, Experiment 1 showed that participants were more successful at ignoring a distractor that signalled a high reward when they were forewarned about the colour of that distractor. Experiment 2 replicated these findings (using an RT-based version of the task) and demonstrated similar effects on performance when participants were provided information about the expected reward on the upcoming trial, rather than the expected colour of the distractor. Finally, Experiment 3 ruled out a potential explanation for these effects – specifically the idea that participants were proactively suppressing attention to the colour of the distractor that they were forewarned would appear.

One prominent account of these data is that orienting to reward-associated distractors in visual search is driven at least in part by a desire to reduce uncertainty and gain information about the current state of the environment (Cogliati Dezza et al., 2023; FitzGibbon et al., 2020; Gottlieb et al., 2020; Gottlieb & Oudeyer, 2018). This active sampling occurs even though participants are aware that it is counterproductive to the goal of earning money in the task. In line with this account, we show here that orienting to a distractor signalling high reward can be virtually eliminated by providing participants with a pre-trial cue as to the type of distractor (or reward) that will appear. This manipulation renders the distractor redundant as a diagnostic source of valuable information, without affecting the relationship between the distractor and the signalled reward.

We did not find any evidence for reduced attentional capture on low-reward trials when participants were instructed as to the upcoming distractor type, relative to non-instructed trials. That is, attention to the low-reward distractor did not decline when pre-trial instruction rendered it redundant as a source of information. Overall, this aspect of the data does not support an information-seeking account where reward and information have independent, additive salience effects (Gottlieb et al., 2020), because some reduced information seeking on low-reward-informative trials should have been measurable, even if reward salience was low. Instead, our data could suggest that reward and information are interacting to dramatically increase salience on high-reward trials only. Overweighting of desirable information, possibly to ‘savour’ knowledge about upcoming reward, has been proposed as a possible motivator for information seeking (Bromberg-Martin & Monosov, 2020; Kobayashi et al., 2019). If information seeking drives attentional prioritisation of distractors in the VMAC task then information about potential high rewards is clearly more valuable (and motivating) than information about potential low rewards, for which information seeking appears to be minimal.

That said, there are some features of our data that are harder to reconcile with the information-seeking account. Surprisingly, in Experiment 1 participants looked *more* frequently at the distractor signalling low reward when they knew to expect it, even though this instruction rendered it entirely predictable and hence redundant as a source of reward information. Furthermore, in Experiment 3, performance improved on trials where the target was unexpectedly rendered in the high-reward colour, relative to all other conditions. If pre-trial information about the upcoming trial type eliminates attentional capture by high-reward distractors because the distractor no longer provides any useful information, then it is hard to explain why a benefit was seen for the target rendered in high-reward colour, even though this also did not provide any extra information.

The information-seeking account described above sees attentional capture as being shaped by the information about reward that distractors provide, with pre-trial instructions modulating this information. An alternative account of the current data proposes instead that: (1) reward-related distractors in the VMAC task capture attention by virtue of their association with reward; and (2) the influence of pre-trial instructions is instead via its motivating influence on the top-down strategy that participants use for search. That is, on high-reward-informed trials participants may have been more motivated to try and use the information about the upcoming distractors to perform a more efficient visual search, since they knew that a high reward was at stake (Konovalov & Krajbich, 2016).

Contrary to the idea of participants strategically using information about upcoming high-reward distractors to improve visual search, we did not find evidence of strategic slowing of eye movements in Experiment 1 when participants knew in advance that high reward would be available relative to when they did not; nor did we find evidence of proactive inhibition of distractor features in Experiment 3. Nonetheless this is by no means an exhaustive assessment of strategies that participants could be using to reduce attentional capture by high-reward distractors in the VMAC task. One possibility is that suppression is reactive rather than proactive. According to the signal-suppression hypothesis, salient distractors generate a bottom-up “attend to me” signal, but rapid recruitment of top-down processes can prevent capture (Gaspelin & Luck, 2018; Sawaki & Luck, 2010). When participants are aware that a high reward is at stake, they may work harder to quickly suppress attention to the salient distractor, which would explain why performance improvements (relative to the generic warning conditions) were observed more readily on high-reward-informative as opposed to low-reward-informative trials. This signal-suppression account could also explain why RT to the target when it was rendered unexpectedly in the high-reward colour was faster than all other conditions in Experiment 3. According to the signal-suppression hypothesis, the high-reward target was salient but top-down suppression was not required (because it was the target rather than the distractor that was salient) and responding was facilitated relative to the low-reward condition because a high reward was at stake. Unlike the information-seeking hypothesis, the motivational account can also explain why participants unexpectedly looked *more* frequently at the low-reward distractor when they knew to expect it, relative to general information trials, in Experiment 1. Because participants knew that only a low reward was available, they did not try very hard to stop themselves looking at the distractor on those trials.

Regardless of the exact mechanism, the current experiments highlight that attentional capture by reward-signalling stimuli is not a foregone conclusion in visual search, which has implications for incentive salience accounts of reward-related attentional capture. These accounts propose that stimuli predictive of reward become themselves imbued with motivational salience via a dopamine-dependent process (Anderson et al., 2016; Berridge, 2007; Colaizzi et al., 2020; Flagel et al., 2007). These stimuli can then cause conditioned Pavlovian approach (“sign tracking”) which is involuntary and persists even if engaging with the stimulus results in the loss of the signalled reward (Hearst & Jenkins, 1974; Watson, Pearson, Wiers, et al., 2019). However, the current finding that attentional capture by high-reward cues virtually disappeared when participants were aware that such a stimulus was forthcoming, suggests that attentional prioritisation of reward-related stimuli is not purely automatic and involuntary. This accords with recent evidence demonstrating that, at least under some conditions, participants are able to prevent themselves from making erroneous saccades to distractors signalling high reward (Grégoire et al., 2022; Pearson & Le Pelley, 2021). For example, Grégoire et al. (2022) demonstrated reduced attentional capture for a reward distractor relative to a neutral distractor, under conditions where only the very fastest responses were rewarded. The authors suggest that the reduced reinforcement ratio provided participants with strong motivation to resist distraction by reward-related stimuli. Using an alternative approach, Pearson and Le Pelley (2021) showed that when a high reward was at stake participants were able to preferentially direct attention to a ‘safe’ distractor and away from the distractor that caused reward omission (see also: Pearson & Le Pelley, 2020). Future research should continue to investigate the interaction between these ‘bottom up’ attentional prioritisation mechanisms and ‘top-down’ control processes, particularly given the relevance of reward-related attentional capture for disorders such as addiction (Watson et al., 2024).

In summary, we showed in three experiments that participants were able to reduce attentional prioritisation of a high-reward stimulus, when they were forewarned of the upcoming distractor type. These results can be interpreted in line with an information-seeking account of VMAC whereby participants orient to the high-reward distractors as a means to gain valuable information about the state of the world, despite this costing them reward. An alternative (not mutually exclusive) interpretation is that participants were able to use the information strategically to improve performance on high-reward trials (possibly by means of improved reactive top-down attentional suppression), which has implications for incentive salience accounts of VMAC. This study offers a novel means to study information seeking in reward-related attentional bias but highlights the difficulties in teasing apart the relative contributions of involuntary Pavlovian reward ‘sign tracking’, information seeking and instrumental behaviour.

## Data Accessibility Statement

All data from this study is freely available at: https://osf.io/w65f2/.
